# Machine Vision Navigation in Spine Surgery

**DOI:** 10.3389/fsurg.2021.640554

**Published:** 2021-03-02

**Authors:** Iain H. Kalfas

**Affiliations:** Cleveland Clinic, Department of Neurosurgery, Cleveland, OH, United States

**Keywords:** image-guided surgery, computer-assisted surgery, spinal navigation, spine surgery, pedicle screws, new technology, innovation

## Abstract

The advancements in computing and digital localizer technologies has led to the evolving clinical application of image-guided technology for the surgical management of spinal disorders. Image-guided spinal navigation addresses the limitations of fluoroscopy and improves the accurate placement of fixation screws. Several navigation platforms are currently available, each having its own unique advantages and disadvantages. The most recent spinal navigation system developed utilizes machine vision structured light imaging which creates a precise and detailed three-dimensional image of the exposed surface anatomy and co-registers it to a pre-operatively or intra-operatively acquired image. This system improves upon the intraoperative workflow and efficiency of the navigation process. With the continued advancements in machine vision, there is a potential for clinical applications that extend beyond surgical navigation. These applications include reducing the potential for wrong level spine surgery and providing for real-time tracking of spinal deformity correction. As the adoption and clinical experience with navigation continues to expand and evolve, the technology that enables navigation also continues to evolve.

## Introduction

The surgical management of spinal disorders has been greatly influenced by the development and use of screw-based fixation devices. The accurate insertion of these screws is critical to ensuring clinical effectiveness and to preventing complications such as neural or vascular injuries or delayed construct failure causing pseudoarthrosis due to improperly positioned screws. Screw insertion accuracy can be achieved to varying degrees by the individual surgeon's spatial and technical skills combined with the use of intraoperative fluoroscopy. Although fluoroscopy has proven to be effective when used to guide screw placement, it also has limitations. It does not provide axial plane imaging which can more accurately demonstrate a screw's medial pedicle breech as opposed to an oblique or antero-posterior (AP) view with fluoroscopy. The image quality can be suboptimal when imaging the upper thoracic region or the lower lumbar region in obese patients. These limitations can lead to varying degrees of screw insertion errors with several studies indicating the rate of disruption of the pedicle cortex by an inserted screw to be as high as 15–31% when using fluoroscopy (1–3).

An additional limitation of fluoroscopy is the radiation exposure experienced by the surgical team and the patient. Rampersaud et al. measured the radiation exposure during lumbar pedicle screw insertion compared to other (non-spinal) orthopedic procedures using intraoperative fluoroscopy and found a 10–12-fold increase in radiation exposure with the lumbar procedures. This added exposure was determined to be due to such factors as the increased energy levels needed to image the lumbar spine as well as to backscatter radiation. Wang et al. compared the mean dosage of radiation exposure delivered by intraoperative fluoroscopy, CT image-guided navigation and robotic assistance in lumbar surgery. Fluoroscopy had the highest radiation dosage (82.02 mGy) followed by robotic assistance (59.84 mGy) and CT image-guided navigation (50.21 mGy). This level of radiation exposure creates a potentially significant hazard to those individuals who perform a high volume of complex spinal surgery with fluoroscopy (4, 5).

Image-guided spinal navigation was developed to address these limitations of fluoroscopy and to improve accurate placement of fixation screws. By combining pre-operative or intraoperative spinal imaging with computer-based localizer technology it creates an interactive three-dimensional (3D) “map” of the spinal surgical anatomy. The display of multi-planar images gives the spinal surgeon a greater degree of orientation and visualization to the otherwise non-visible spinal anatomy. This facilitates an improvement in screw insertion accuracy. It also eliminates the need for conventional fluoroscopy and reduces radiation exposure to the surgical team (6, 7).

## History of Navigation in Spine Surgery

The development of image-guided spinal navigation in the early 1990's was based on the principles of stereotaxy which is defined as a three-dimensional coordinate system to locate specific points in space. Stereotactic techniques have been used for several decades primarily for the intraoperative localization of intracranial pathology. Earlier techniques of stereotactic surgery required the use of a rigid frame attached to the patient's head. The frame was in place for both pre-operative imaging and for the surgical procedure serving as an external reference point linking the image data to the surgical anatomy. While the use of a frame was feasible for intracranial surgery it was impractical for other surgical procedures.

With the advancements in computing and digital localizer technologies in the late 1980's, stereotactic techniques were further developed and could be performed without an attached frame. In addition to improving accuracy for cranial surgery, “frameless” navigation technology now allows for the application of stereotactic techniques to extracranial procedures, particularly, spinal surgery. The increasing popularity of pedicle screw fixation in the early 1990's led to a growing interest in frameless techniques which ultimately led to the development of image-guided spinal navigation.

The initial application of image-guided technology to spinal surgery was reported by Kalfas and colleagues in 1994 and 1995 (6, 7). These reports demonstrated the feasibility of using navigational technology to improve the accuracy and efficiency of lumbar pedicle screw insertion without the need for intraoperative imaging. The effectiveness of navigation for pedicle screw insertion was further demonstrated by other groups as adoption of the technology progressed (8–12). Other applications of navigation to spinal surgery evolved including cervical screw fixation, transoral decompression, cervical corpectomy, anterior thoracolumbar fixation and placement of iliac screws (13–17). Navigation was also successfully integrated to minimally invasive spinal surgery to reduce the significant radiation exposure that occurs with these procedures when using conventional fluoroscopy (18, 19).

The continued development of optical localizers, faster computing speeds, intraoperative computed tomography (CT) imaging and surgical robotics coupled with an expanded clinical experience has allowed the technology to improve and evolve into what is now a well-accepted and commonly used surgical support technology. This evolution of spinal navigation has led to the development of several different techniques and navigation systems. The first navigation system successfully applied to spinal surgery was pre-operative CT-based navigation (6, 7). Other navigation options soon evolved including fluoroscopic navigation, isocentric fluoroscopic navigation, intraoperative CT-based navigation, and robotic navigation. Each of these techniques offered unique advantages and disadvantages when compared to the others but they all provided an improvement in screw insertion accuracy and a reduction in radiation exposure when compared to conventional fluoroscopy (20, 21).

## Machine Vision Navigation Technology

The most recent spinal navigation system developed utilizes a technology termed machine vision. This technology couples video cameras with computer systems to obtain and process images for a variety of applications. Machine vision has been used for several years in numerous industries for applications, such as automated inspection, process control, robotic guidance, facial recognition and self-driving vehicles. Its application for spinal surgery navigation uses a specific type of machine vision termed structured light imaging. Combining a light projector with two stereoscopic video cameras, this version of machine vision captures a precise and detailed three-dimensional image of the exposed surface anatomy and co-registers it to a pre-operatively or intra-operatively acquired image (e.g., fluoroscopy, CT) data set. This application of machine vision has been successfully applied to both cranial and spinal surgery.

**Figure 1:**
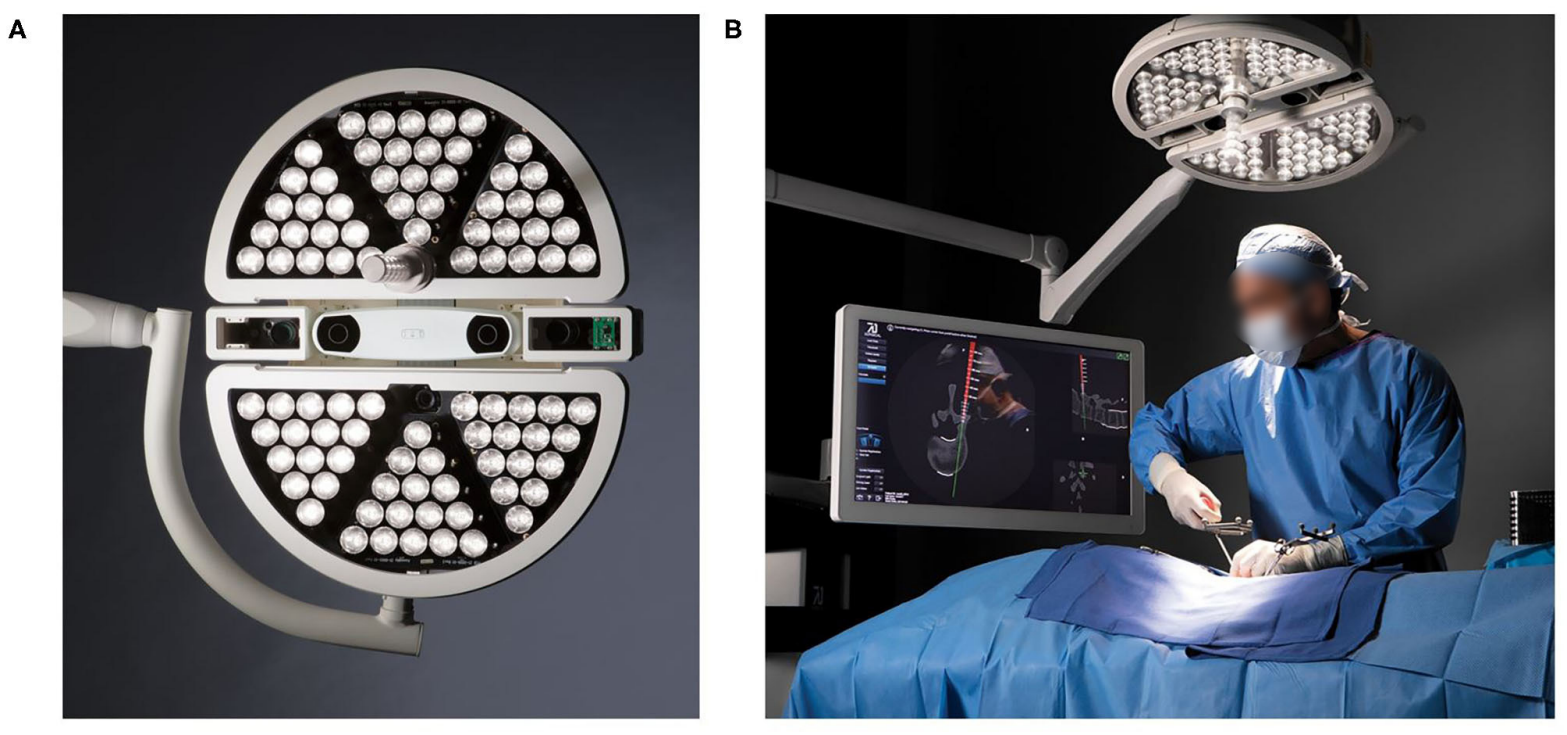
The 7D Surgical System surgical light head **(A)** contains the navigation optics as well as LED lights used for standard lighting. The navigation optics include binocular infrared cameras for tool tracking, stereovision video cameras and projector for machine vision and a structured light projection system. **(B)** The 7D Surgical navigation system used in clinical setting.

The 7D Surgical System is the first machine vision navigation system designed for spine surgery. It consists of a small, mobile computer workstation with an attached, movable arm (Figure 1). The arm is connected to the system head which consists of a surgical light source, two stereoscopic video cameras, a structured light projector, and an infrared camera system for tracking navigation tools.

The navigation workflow for the 7D Surgical System is initiated by loading a pre-operatively acquired CT data set of the appropriate spinal levels on to the system's workstation. If a pre-operative CT is not available, an intra-operatively acquired CT date set can be used. The workstation is positioned adjacent to the surgical table and the arm adjusted to place the system head directly over the surgical field. The surgical lamp of the system head provides sufficient illumination of the field reducing the need for the standard ceiling mounted surgical lights. If the standard lights are used during the surgical exposure, they need to be directed away from the field during the navigation process because of their potential to interfere with the structured light imaging process.

Following surgical exposure and attachment of a dynamic reference array in the field, the selected navigation tools are registered. The system head is then accurately aimed toward the surgical field with the aid of laser guidance. Two stereoscopic video images of the field are displayed on the workstation monitor. When activated, the structured light projector in the system head briefly projects a linear light grid pattern onto the surgical field. As this occurs, the three-dimensional surgical anatomy distorts the lines of the light pattern. The degree of this distortion is detected by the overhead stereoscopic video cameras. The color system captures > ~1,000,000 data points over a 40 × 30 cm surface area, yielding a resolution of 4–6 points per square millimeter (22). The specific distortion of the light pattern is then used to calculate surface depths in order to reconstruct the three-dimensional topography of the exposed surgical surface anatomy (22). This reconstructed image data set is then rapidly co-registered to the pre-operative CT scan and the navigation process can proceed.

A significant advantage of machine vision registration compared to other navigation systems is that it requires only seconds to complete and can be easily repeated (22, 23). The need to repeat the registration process typically occurs when the reference array has been purposely or inadvertently moved. It may also be necessary to re-register when navigating several levels away from the site of the reference array to ensure accuracy. Uehara et al. have demonstrated that the accuracy of navigational date decreases when navigating greater than three levels away from the site of attachment of the array (24). With intraoperative CT or fluoroscopic navigation, repeating the registration process requires repeating the imaging process. This involves bringing the imaging system back into the room, re-positioning it and obtaining updated images which adds significant time and radiation exposure to the procedure.

With the 7D Surgical System, repeating the registration process requires only an additional projection of the light pattern grid onto the surgical field with the re-positioned reference array. The updated image reconstruction is then re-registered to the stored image data set and navigation can resume. As with the initial registration, this process takes only seconds to repeat. This ability to rapidly re-register allows for each vertebrae that is being instrumented to be individually registered to optimize navigational accuracy (22, 23).

Following registration, the navigation process with the 7D Surgical System is similar to other systems. Tools are trackable using infrared cameras in the system and include an awl, a pedicle probe, and a drill guide. Multiplanar images are projected in real time indicating the selected entry points and trajectories through the spinal anatomy. Pilot holes through the selected trajectories can be navigated and drilled followed by freehand placement of the screws. Alternatively, a universal tracking device can be attached to any tap or screwdriver if visual navigation of screw insertion is preferred. During screw insertion, no real-time imaging (i.e., fluoroscopy) is required. A lateral plain film radiograph is obtained at the start of the surgery to confirm level localization and immediately prior to wound closure to document baseline screw placement.

**Figure 2:**
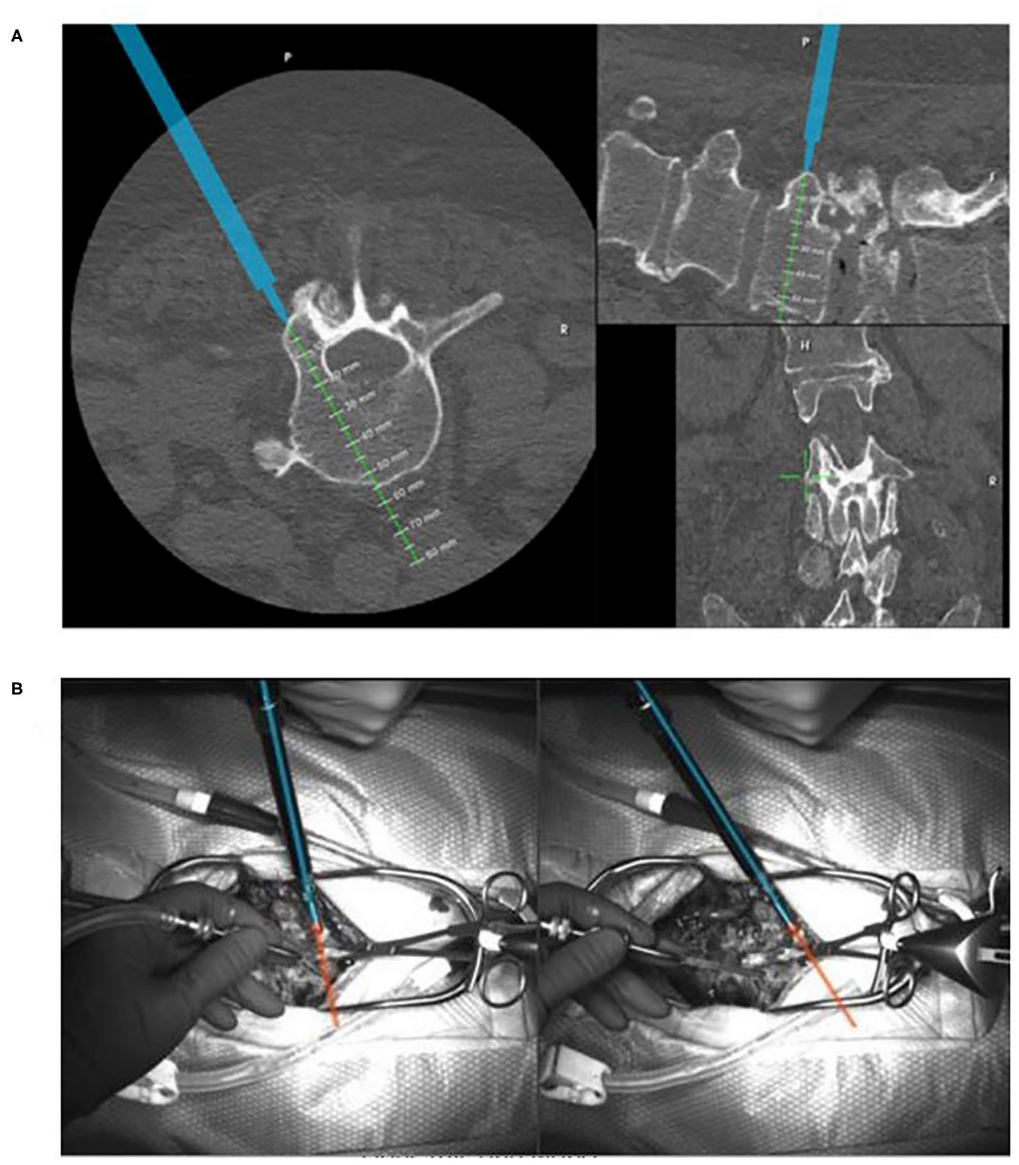
Display of the navigation screen of the 7D Surgical System **(A)** showing the tracked tool in blue and the intended trajectory in green. The surgeons overhead view of the standard surgical site **(B)** with the trajectory created by AR displayed.

An additional unique feature of the 7D Surgical System is the option of using augmented reality (AR) to facilitate accuracy and safety. Nguyen et al. demonstrated that AR software can be added to machine vision navigation to superimpose “safe” zones and trajectories through selected pedicles (25). When a navigated trajectory has been selected, a virtual line along that trajectory can be projected onto the system's video monitor and preserved during screw insertion (Figure 2). This is particularly helpful under certain circumstances when tool tracking is not feasible. Aligning a non-tracked tap or screwdriver with the virtual line on the live video feed preserves the screw insertion accuracy (26).

The initial clinical experience with the 7D machine vision navigation system has demonstrated that it significantly improves upon the intraoperative workflow and efficiency of the navigation process when compared to other navigation options. It provides the same level of screw insertion accuracy in less time and without the need for any intraoperative imaging or radiation exposure (27).

The current price of the 7D Surgical System is ~470,000 USD. The price compares favorably to other navigation platforms in that it is approximately one third the price of an intra-operative CT-based navigation system. The only disposables associated with the system are the single use reflective spheres that are attached to the reference array and navigation tools which come with the system.

## Clinical Applications

As with other spinal navigation systems, the most common clinical application of machine vision navigation is for lumbosacral and thoracic pedicle screw insertion. It can also be used for the insertion of iliac screws and posterior cervical screws. A prospective clinical study of 171 cranial and spinal surgical procedures compared the 7D Surgical System to two legacy navigation systems specifically assessing fixation screw placement accuracy and registration workflow time. Analysis of 162 thoracic, lumbar, and cervical fixation screws found no significant differences in breach rates, angular error, and translational error between the machine vision system and the benchmark navigation systems. The advantage of the 7D Surgical System in this study was that it consistently demonstrated a more rapid setup and registration time (41 s vs. 258 and 794 s) compared to the two other systems confirming its workflow efficiency (22).

Another prospective clinical study assessed the accuracy of machine vision navigation for the placement of fixation screws during posterior cervical surgery. Seventy-four cervical fixation screws in fifteen patients were placed using the 7D Surgical System. Additionally, fifty-three cervical screws were placed into four cadaver cervical spines. The study found screw insertion accuracy that was comparable to other navigation systems (28).

The 7D Surgical System has also been evaluated for use with midline mini-open spinal procedures. A prospective clinical (*n* = 8 patients, 55 screws) and cadaveric (*n* = 4 cadavers, 37 screws) study investigated the accuracy of the 7D Surgical System with midline exposures ranging from 25 to 40 mm. The screw insertion accuracy when using the 7D Surgical System with a mini-open approach was comparable to the accuracy in open procedures (29).

The primary disadvantage of the 7D surgical system compared to other navigation platforms is that, in its current design, it is not capable of providing navigation for percutaneous screw insertion. This is due to the fact that the structured light projection used by machine vision technology requires some degree of visualization of spinal surface anatomy in order to achieve accurate registration. Currently, navigation for percutaneous screw insertion requires a navigation platform that uses intraoperativley acquired fluoroscopic or CT images to navigate screw insertion. Despite this disadvantage, further design and development to enable the 7D surgical system to provide percutaneous screw navigation has been carried out and the resultant technology is currently under regulatory review for approval. Unlike the application of the system to open procedures, this feature will require obtaining intraoperative CT imaging.

## Future Directions

The optical technologies and algorithms employed by machine vision navigation allow for potential clinical applications that extend beyond surgical navigation. One of these clinical applications includes mitigating wrong level spinal surgery that has a reported incidence ranging from 1 in 2,222 to 1 in 3,010 with 50% of surgeons performing a wrong level spine surgery at some point during their career (30). Zagzoog et al. described a method that uses the machine vision image guidance system's structured light images and software algorithms to detect wrong level spine surgery. The study tested four detectors of wrong level spine surgery using 310 measurements from machine vision image data. Each detector was able to determine if an indicated spine level was correct with 94% accuracy (31). These results indicate a potential for integrating these detectors into a machine vision system to provide real-time feedback during surgery that can verify the correct spinal level and reduce the incidence of wrong level surgery.

Another feature of machine vision technology being investigated is the ability to detect and display intraoperative changes in spinal alignment during spinal deformity surgery. The system's software utilizes advanced object recognition and registration algorithms that enable segmental registration of the individual vertebrae from the pre-operative CT data set. Individual virtual models of each vertebra can be created, and their relative location measured. Following correction of a spinal deformity during surgery, the registration process is repeated capturing the exposed surface anatomy in its new position. The virtual models of the involved vertebrae are displayed in their re-aligned positions providing the surgeon with real-time feedback regarding the degree of achieved correction. The system also provides Cobb angle measurements that can indicate if further coronal or sagittal correction is required. If additional correction is performed, repeating the registration process will update the virtual deformity correction image.

## Discussion

Despite the supportive clinical evidence and technical advances made over two decades of use, the adoption of spinal navigation has been relatively slow and inconsistent (32). A critical barrier to adoption continues to be the high cost of the technology, particularly outside of the United States. Reimbursement for the use of navigation is relatively small. In addition to the initial capital expense for purchase, the additional costs for these systems include the development and training of surgical support staff, the use of disposable instruments and the potential for added surgical time in the early learning stages of the procedure. Efforts to justify and optimize the cost vs. value proposition of spinal navigation are generally focused on the objective of reducing or eliminating the need for revision surgery due to screw misplacement and limiting any associated medico-legal risk exposure. This has been demonstrated to be practical in centers that perform a high volume of complex spinal deformity procedures (33).

Another factor that has limited the widespread adoption of spinal navigation is its variable interference with the normal workflow of the procedure. Early navigation systems routinely increased the overall operative time due to difficulties with manual registration, system (software) failures, inadequate navigation tools and lack of trained support personnel. Current barriers to efficient workflow with navigation include the added time needed to obtain an intraoperative CT, a limited number of levels visualized on the intraoperative CT, the need to repeat imaging if the reference array in the surgical field is moved, the lack of a simple, repeatable registration process and the difficulties associated with maintaining a line of sight between the localizer camera and the navigated instruments.

The development of machine vision technology addresses and mitigates each of these workflow issues. The image data used is acquired pre-operatively eliminating the time needed for intraoperative imaging. All the levels to be instrumented are visualized as opposed to the field of view limitations associated with some intraoperative imaging systems. The registration process is rapid and repeatable. Inadvertent movement of the attached dynamic reference array does not require taking additional time to repeat the imaging process to re-register the spinal anatomy as is the case with navigation systems that uses intraoperative imaging. With machine vision navigation, the reference array can be re-positioned, and a repeat registration process performed in seconds. This also allows for intentional re-positioning of the reference array in cases of multi-level instrumentation to improve the accuracy of navigational trajectory information. Each of these features contribute to intraoperative workflow efficiency and help reduce the time needed to perform spinal navigation.

## Conclusion

Image-guided spinal navigation has become an accepted and proven surgical technology for improving the insertion accuracy of spinal fixation screws. As the adoption and clinical experience with navigation has expanded, the techniques and technical components have also continued to evolve. Several different navigation platforms are currently available, each having its own unique advantages and disadvantages but each providing a level of accuracy greater than conventional fluoroscopy. Machine vision navigation is the most recent iteration of this technology. It provides the same level of accuracy as other navigation systems but with greater intraoperative workflow efficiency and without the need for intraoperative radiation.

## Author Contributions

IK provided substantial contributions to the concept and design of the work, the involvement in retrieving conceptual information and draft manuscript preparation, and performed critical revision to structure the content intellectually and gave approval for the final version to be published.

## Conflict of Interest

IK declares that he is a consultant for 7D Surgical.
